# A Comparison of Community Resources for Chinese Immigrants Across Philadelphia Neighborhoods

**DOI:** 10.5888/pcd18.200615

**Published:** 2021-04-15

**Authors:** Ava Wagner, Vikram Bellamkonda, Russell White, Emily Walton, Carolyn Y. Fang, Marilyn Tseng

**Affiliations:** 1 Fourth-year Bachelor of Science candidate, California Polytechnic State University, San Luis Obispo, California; 2 Robert E. Kennedy Library, California Polytechnic State University, San Luis Obispo, California; 3 Department of Sociology, Dartmouth College, Hanover, New Hampshire; 4 Cancer Prevention and Control Program, Fox Chase Cancer Center, Philadelphia, Pennsylvania; 5 Department of Kinesiology and Public Health, California Polytechnic State University, San Luis Obispo, California

**Figure:**
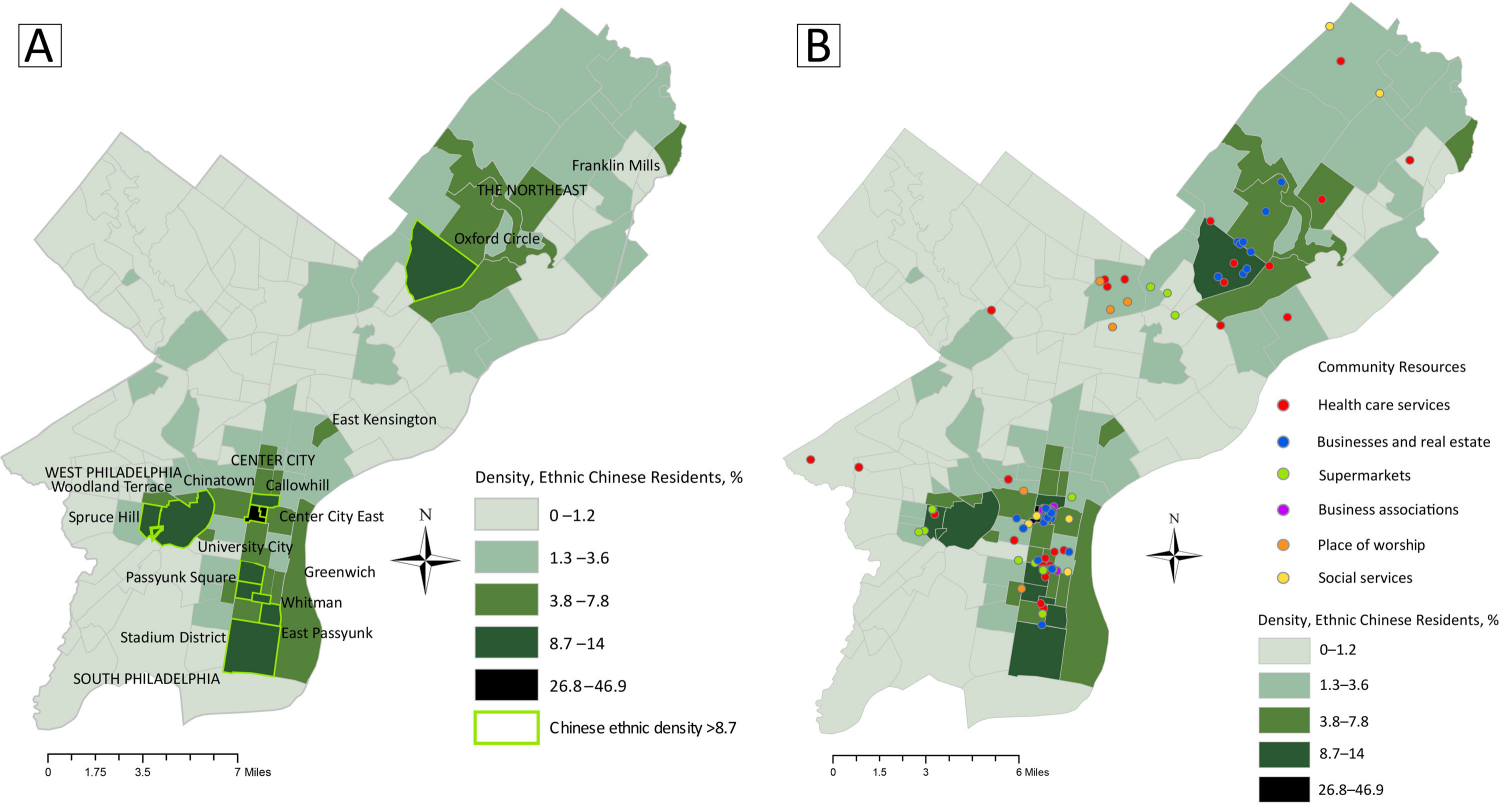
Distribution of neighborhoods and services in Philadelphia, Pennsylvania, by density of ethnic Chinese residents, from 2014–2018 estimates. Map A shows percentages by neighborhood, highlighting those with a density of 8.7% or more. Map B shows locations of 6 types of community resources for Chinese residents overlaid on Map A to illustrate resource distribution in relation to population density. Geographic proximity of resources corresponds overall to neighborhood density of Chinese residents. However, not all types of resources are equally distributed, indicating they are unavailable to residents of some neighborhoods. Data sources: Chinese demographic data are from the American Community Survey 2018 (5-Year Estimates), prepared by Social Explorer (1). Boundaries for Philadelphia neighborhoods data are from OpenDataPhilly, developed by Azavea Inc (2). Community resource data are from the Chinese *Philadelphia Yellow Pages* (3).

## Background

Chinese immigrants are the third-largest non-US–born population in the US (4). Although these immigrants have lower rates of obesity and obesity-related conditions than people of other races/ethnicities, such health advantages decrease with increasing length of US residence (5,6). This increased risk for chronic disease has been attributed primarily to acculturation to Western lifestyle norms; however, trajectories may vary depending on the environment in which immigrants reside (7). Ethnic enclaves are ethnically, spatially, and socially distinctive communities with sizable immigrant populations that have been shown to promote well-being with their concentration of health-related, cultural, and social resources (8). Such resources include health care providers and retail food stores that share their language and culture, and gathering spaces for social interactions, such as churches (9,10). Research among elderly immigrants has shown that the incorporation of Chinese cultural symbols in the physical spaces they inhabit increases immigrants’ sense of belonging (11). Access to cultural resources, such as places of worship and schools, may also yield tangible health benefits over time (12). The interpersonal connections, social networks, and sense of cohesion and belonging fostered in environments that are socially and culturally resource-rich may increase Chinese residents’ social capital, which has been associated with a wide range of positive health outcomes, including reduced risk for chronic disease (10,13).

The Philadelphia metropolitan area is among the top 10 destinations for Chinese immigrants to the US. Of the approximately 37,000 ethnic Chinese people who resided in Philadelphia according to 2014–2018 estimates, 60% were non-US–born (1). The availability and locations of various types of community resources in neighborhoods of high ethnic Chinese density could help direct immigrants toward the resources they need and help determine where resources are still needed. However, such information is largely unavailable. As part of a study of Chinese immigrants residing in Philadelphia, we mapped the spatial distribution of 8 types of health-related Chinese community resources, overlayed on the density of Chinese residents in neighborhoods across the city. Our objective was to show the relative proximity of such resources to the neighborhoods with high concentrations of ethnic Chinese residents and areas with high density but few resources.

## Data and Methods

To identify community resources for the largely non-US–born Chinese population residing in Philadelphia, we used the most current online Chinese version of the *Philadelphia Yellow Pages* (3). We further investigated these resources by using Google searches to verify that they targeted Chinese clients through Chinese-language advertising or other information. We then categorized each as one of 6 types of resources: primary health care provider (n = 46) (ie, family medicine, internal medicine, pediatrics, Chinese medicine, dentistry), places of worship (n = 14), business and cultural associations (n = 16), supermarkets (n = 29), other businesses (n = 43) (ie, accounting, insurance, banks, real estate), or other services (n = 10) (ie, employment, funeral, English language, education). The address of each resource was geocoded and color-coded, then mapped as a layer in ArcGIS 10.8 (ESRI). Each resource map was then overlaid on a map showing neighborhoods by density of ethnic Chinese residents.

We defined geospatial neighborhood boundaries by using a web map (2) of Philadelphia neighborhoods. We used 2018 American Community Survey 5-year estimates to calculate census tract–level density of ethnic Chinese residents as the number of people of Chinese origin, excluding Taiwanese people, divided by the total population of the census tract (1). The census tract–level data were aggregated within neighborhood boundaries according to the proportion of their spatial areas that fell within the boundaries. For example, a census tract that fell completely within a given neighborhood was included in its entirety, but for a census tract that fell only halfway within a given neighborhood, only 50% of its population was included. We categorized ethnic Chinese density in 5 ranges (0%–1.2%, 1.3%–3.6%, 3.8%–7.8%, 8.7%–14.0%, and 26.8%–46.8%) by using the Jenks method, which identified natural breaks in the distribution of ethnic density. We used a grayscale to illustrate the levels of ethnic density. We designated neighborhoods in the top 2 categories (>8.7%) as having high ethnic Chinese density.

## Highlights

Across 157 Philadelphia neighborhoods, 3 contiguous neighborhoods in Center City had the highest concentrations of ethnic Chinese residents: Chinatown, 46.9%; Center City East, 26.8%; and Callowhill, 14.0%. They were followed by 3 clusters of adjacent neighborhoods: South Philadelphia (Greenwich, 13.4%; Passyunk Square, 12.7%; East Passyunk, 12.0%; Stadium District, 9.9%; Whitman, 9.1%), West Philadelphia (Spruce Hill, 11.1%; University City, 9.1%; Woodland Terrace, 8.7%), and the Northeast (Oxford Circle, 10.1%). Community resources were heavily concentrated in these 4 areas. In particular, of the 158 resources that we mapped, 76 (48.1%) were located in the 3 Center City neighborhoods centered on Chinatown — mostly supermarkets, businesses, and business and cultural associations. These 3 Center City neighborhoods were the only ones that also contained all 6 resource types, primarily because 15 of the 16 business and cultural associations were located in these neighborhoods.

Chinese-speaking health care providers, although concentrated in Chinatown and near South Philadelphia, were widely distributed across the city, even in areas of low ethnic Chinese density. In contrast, businesses were concentrated in Center City, South Philadelphia, and Oxford Circle, although Oxford Circle did not have supermarkets. The West and South Philadelphia neighborhoods and Oxford Circle had fewer places of worship; places of worship in the Northeast were located in the neighborhood of Olney, which is southwest of Oxford Circle. The neighborhoods of Franklin Mills and East Kensington lacked any resources despite their relatively high ethnic density (7.5% and 7.4%, respectively).

## Action

Our maps have 2 primary implications for preventing chronic disease among Chinese immigrants. First, they help identify neighborhoods of high ethnic Chinese density with few nearby culture-specific resources, and they highlight the specific types of resources that are lacking. As such, the maps can complement needs assessments targeting neighborhoods with Chinese immigrants to determine the types of resources that might be fostered in these areas. In turn, needs assessments can inform future iterations of these maps by incorporating additional types of resources and ways to categorize these resources. Second, the maps inform Chinese immigrants in these areas who might not be aware of the full range of social and cultural resources in the city beyond their immediate neighborhoods.

These maps also suggest that Chinese immigrants do not limit themselves to the resources in their immediate residential environment. This idea of “heterolocality” (14) points to the importance of studying how immigrants navigate their environments to meet their social and cultural needs and preferences.
